# Acetylation modification in malignant progression and therapeutic resistance of gliomas

**DOI:** 10.3389/fcell.2026.1808361

**Published:** 2026-03-25

**Authors:** Jiayi Zhang, Mengying Wang, Hong Wang, Xuemei Jiang, Hanyan Zou

**Affiliations:** Chongqing Institute for Food and Drug Control, CQMPA Key Laboratory for Development and Evaluation of Innovative Biological Products, Chongqing, China

**Keywords:** acetylation, glioma, inhibitor, malignant progression, therapeutic resistance

## Abstract

Gliomas are the most common and aggressive primary malignant tumors of the central nervous system, characterized by diffuse infiltration, rapid proliferation and dismal prognosis. Despite advances in multimodal treatments, including surgical resection, radiotherapy and targeted therapies, clinical outcomes remain unsatisfactory owing to the inevitable development of therapeutic resistance. Protein acetylation, a key post-translational modification dynamically regulated by lysine acetyltransferases and deacetylases, plays a central role in governing chromatin organization, transcriptional regulation, protein stability and signal transduction. Increasing evidence indicates that aberrant acetylation critically contributes to glioma malignant progression and treatment resistance by modulating essential cellular processes, including proliferation, apoptosis, invasion, metabolic reprogramming, DNA damage repair, immune evasion and cancer stemness. This review systematically summarizes recent advances in understanding the roles of both histone and non-histone acetylation in glioma biology, with particular emphasis on their involvement in resistance to chemotherapy and radiotherapy. Collectively, this review underscores acetylation as a pivotal epigenetic mechanism driving glioma aggressiveness and therapeutic resistance and provides mechanistic insights and a conceptual framework for the development of more effective and precise therapeutic strategies.

## 1 Introduction

Gliomas originate from glial cells or neural stem/progenitor cells in the central nervous system (CNS) and account for approximately 80% of all primary malignant brain tumors (Price et al., 2025; Singh et al., 2004). According to the World Health Organization (WHO) classification of CNS tumors (2021 edition), gliomas are classified into grades I to IV based on their histological features and biological aggressiveness. Low-grade gliomas (WHO grades I–II) typically exhibit slow growth but tend to progress to high-grade gliomas over time. Among high-grade gliomas, glioblastoma (WHO grade IV) is the most aggressive subtype; even with the incorporation of tumor treating fields (TTFields) into standard therapy since 2016, its median overall survival (mOS) remains only 20.9 months (Louis et al., 2021; Stupp et al., 2017). The poor prognosis of gliomas is mainly attributed to their unique biological characteristics, including extensive invasiveness into the surrounding normal brain tissue, which makes complete surgical resection impossible, high proliferative capacity and the ability to evade immune surveillance (Louis et al., 2021; Peng et al., 2022; Wu et al., 2025). Most patients experience rapid tumor recurrence after treatment and subsequently develop resistance to chemotherapeutic agents, which markedly limits therapeutic efficacy. Consequently, glioblastoma remains one of the most challenging diseases in contemporary neuro-oncology and overcoming therapeutic resistance while improving patient survival represents an urgent and unresolved clinical challenge.

In recent years, accumulating evidence has highlighted epigenetic dysregulation as a central driver of glioma initiation, progression, recurrence and therapy resistance (Pienkowski et al., 2023; Dong and Cui, 2019). Unlike genetic alterations, which are irreversible and fixed, epigenetic modifications regulate gene expression and signaling pathway activity without altering the underlying DNA sequence (Pan and Chen, 2022). Epigenetic mechanisms including DNA methylation, histone modifications, chromatin remodeling and non-coding RNAs that govern glioma cell proliferation, invasion, stemness, immune escape and resistance to therapy (Ilango et al., 2020). Among these regulatory ways, post-translational modifications (PTMs) of proteins have emerged as particularly dynamic and versatile modulators of tumor behavior. Protein PTMs refer to enzymatically catalyzed covalent modifications that occur after protein synthesis, involving the addition or removal of chemical groups at specific amino acid residues. These modifications profoundly influence protein conformation, stability, subcellular localization, enzymatic activity and protein–protein interaction networks, thereby fine-tuning cellular signaling pathways and transcriptional functions (Le et al., 2023). To date, more than 600 distinct PTM types have been identified, including phosphorylation, acetylation, ubiquitination, sumoylation, methylation, glycosylation, palmitoylation and succinylation (Walsh et al., 2005). Dysregulation of PTMs disrupts cellular homeostasis and has been extensively implicated in cancer pathogenesis. In gliomas, aberrant PTM coordinate oncogenic signaling, promote malignant phenotypes and confer resistance to conventional and targeted therapies (Haase et al., 2024).

Among diverse PTMs, lysine acetylation has emerged as one of the most extensively studied and functionally significant epigenetic modifications in cancer (Kaypee et al., 2016). Acetylation is dynamically regulated by lysine acetyltransferases (KATs) and lysine deacetylases (HDACs), which respectively catalyze the addition and removal of acetyl groups. By adding acetyl groups to the lysine residues, acetylation alters protein structure and interaction capacity, exerting profound effects on chromatin organization, transcriptional regulation, protein stability and signal transduction. Historically regarded as a histone-specific modification, acetylation is now recognized to extensively modify non-histone proteins, including transcription factors, signaling mediators, metabolic enzymes and DNA repair proteins, thereby expanding its functional repertoire far beyond chromatin regulation. Notably, previous studies have demonstrated that histone H3 acetylation is broadly upregulated in gliomas compared with normal brain tissue. Importantly, excessive elevation of histone acetylation levels has been identified as a key driver of malignant progression in high-grade gliomas (Yu et al., 2014; Huang et al., 2015; Herz et al., 2014; Lucio-Eterovic et al., 2008). Aberrant histone acetylation promotes chromatin relaxation and transcriptional activation of oncogenes and stemness-associated factors, facilitating uncontrolled proliferation, invasive growth and resistance to therapy (Hou et al., 2017; Tang et al., 2025; Chen et al., 2025). In parallel, dysregulated acetylation of non-histone proteins directly modulates the activity, stability and localization of critical oncogenic regulators, thereby reinforcing malignant signaling networks (Zhang et al., 2025; Kunadis et al., 2021). Emerging evidence further implicates acetylation as a pivotal mediator of therapeutic resistance in gliomas (Clarke et al., 2013). Acetylation-dependent regulation of DNA damage repair pathways, cell-cycle checkpoints and apoptotic signaling critically influences glioma cell sensitivity to TMZ and radiotherapy (Chen et al., 2025). For instance, acetylation-driven transcriptional activation of DNA damage repair–related genes enhance the ability of glioma cells to withstand therapeutic stress, while acetylation-mediated stabilization of oncogenic transcription factors sustains survival signaling under therapeutic pressure (Miao et al., 2025; Dang and Wei, 2022). Moreover, emerging evidence indicates that aberrant acetylation plays a critical role in maintaining glioma stem cell properties and facilitating immune evasion, thereby jointly promoting tumor recurrence and the development of therapeutic resistance (Va et al., 2020; Qin et al., 2024).

In this review, we provide a comprehensive overview of recent advances in understanding the roles of protein acetylation in glioma malignant progression and therapeutic resistance. We further highlight emerging therapeutic strategies targeting acetylation-related regulatory pathways, discuss current challenges in this field, and propose future directions for translating acetylation-based mechanistic insights into more effective and precise therapeutic interventions for glioma patients.

## 2 Overview of protein acetylation

Protein acetylation is a major post-translational modification (PTM) in eukaryotes, involving the transfer of acetyl groups to specific sites on polypeptide chains. This process is catalyzed by various acetyltransferases and has been increasingly recognized as a complex and systematic regulatory mechanism with the advancement of proteomic analysis and functional research in recent years.

The regulation of protein acetylation relies on two major classes of enzymes. KATs are classified into Type A and Type B based on their subcellular localization and function. Type A KATs, primarily nuclear, catalyze acetylation of histones and non-histones involved in transcriptional regulation, while Type B KATs, localized in the cytoplasm, acetylate newly synthesized free histones to facilitate nuclear translocation (Choudhary et al., 2014; Glozak et al., 2005; Allis et al., 2007; Arrowsmith et al., 2012). Key KAT families include p300/CBP (catalyzing 80%–90% of histone and non-histone acetylation events) (He et al., 2021; Berndsen and Denu, 2008), MYST (with conserved catalytic domains, e.g., Tip60, KAT7) (Li et al., 2021) and GNAT (evolutionarily conserved, e.g., Gcn5, PCAF) (Dyda et al., 2000; Salah Ud-Din et al., 2016). Deacetylases are divided into zinc-dependent HDACs (Classes I-IV) and NAD + -dependent sirtuins (Class III) (Leite et al., 2022). HDACs regulate chromatin state and gene transcription—acetylation relaxes chromatin to promote transcription, while deacetylation has the opposite effect (Wan et al., 2008).

**FIGURE 1:**
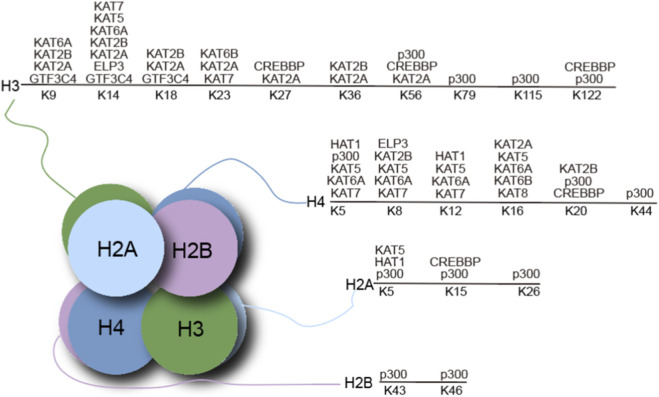
Schematic diagram of related acetyltransferases and their histone substrates.

Protein acetylation exerts profound and far-reaching effects on numerous essential cellular processes, serving as a key regulatory switch in cell physiology. Histone acetylation, which primarily occurs at specific lysine residues in the N-terminal tails of histones H3 and H4, modulates transcriptional activity by reducing the electrostatic attraction between positively charged histone tails and negatively charged DNA, thereby loosening the interaction between histones and DNA (Espinola-Lopez and Tan, 2021). Notably, histone acetylation is not restricted to a limited number of residues but instead occurs at numerous lysine sites across core histone proteins, forming a complex and highly dynamic modification landscape (Bilinski et al., 2012). Histones H3, H4, H2A, and H2B harbor multiple acetylation sites that are catalyzed by distinct lysine acetyltransferases. These include several well-characterized residues, such as H3K9, H3K14, H3K18, H3K27, H3K36, H3K56, H3K79, H3K115 and H3K122; H4K5, H4K8, H4K12 and H4K16; as well as H2AK5, H2AK15, H2AK26, H2BK43 and H2BK46 (Figure 1). These distinct acetylation marks can act individually or in combination to regulate chromatin structure and transcriptional function. The site-specific and combinatorial nature of histone acetylation enables precise regulation of gene expression programs in response to developmental cues, environmental stimuli, and cellular stress, highlighting its central role in epigenetic control.

Non-histone acetylation, on the other hand, regulates a diverse array of cellular functions through modifying various substrate proteins (Table 1). For example, it participates in gene transcription regulation by acetylating transcription factors such as p53 and STAT3, which modulates their DNA-binding affinity and transcriptional activity (Narita et al., 2019); it controls cell cycle progression by targeting key cyclin-dependent kinases (e.g., CDK1, CDK2) and checkpoint proteins (e.g., BUBR1) (Choi et al., 2009; Mateo et al., 2009; Xu et al., 2023); and it facilitates DNA repair processes by activating kinases such as ATM and promoting the recruitment of DNA repair factors (e.g. 53BP1) to DNA damage sites (Guo et al., 2018). Dysregulation of protein acetylation is closely associated with the development and progression of various diseases, particularly cancer. Abnormal expression of KATs and KDACs often leads to aberrant acetylation patterns that drive tumorigenesis: for instance, abnormal expression of p300/CBP is linked to tumor progression by dysregulating the acetylation of oncogenes and tumor suppressor genes (Iyer et al., 2004; Welti et al., 2021; Sauer et al., 2017); KAT7 promotes the malignant progression of liver, breast and gastric cancers by activating oncogenic pathways such as PI3K/AKT and Wnt/β-catenin through targeted acetylation (Ma et al., 2023; Wang et al., 2023); and GCN5 contributes to the progression of breast cancer and non-small cell lung cancer by regulating epithelial-mesenchymal transition (EMT) and c-Myc activity *via* histone acetylation (Mustachio et al., 2019; Zhao et al., 2018). Consequently, KAT and HDAC inhibitors have emerged as promising therapeutic agents for cancer treatment. Several HDAC inhibitors have been approved by the U.S. Food and Drug Administration (FDA) for the treatment of various types of tumors, and numerous KAT inhibitors are currently under preclinical and clinical evaluation (He et al., 2021; Welti et al., 2021; McClure et al., 2018; Zhang et al., 2019).

**TABLE 1:** Lysine acetyltransferase and its non-histone substrate.

Acetyltransferase	Non-histone substrate
p300/CBP	p53 (Grossman, 2001), EKLF (Dempsey et al., 2003), STAT6 (Gingras et al., 1999), RELA (Buerki et al., 2008), E2F1 (Manickavinayaham et al., 2019), AR (Xu et al., 2025), KPNA2 (Feng et al., 2023), MyoD (Polesskaya et al., 2001), SP3 (White et al., 2006), TDG (Mohan et al., 2010), CART1 (Iioka et al., 2003), STAT3 (Wang et al., 2017a), SMAD2 (Inoue et al., 2007), SP1 (Camolotto et al., 2013), BACE1 (Iyer et al., 2004), FLI1 (Lennard et al., 2016), EVI1 (Shimahara et al., 2010), WRN (Li et al., 2010), GATA1 (Boyes et al., 1998), HBP1 (Wang et al., 2012), SPK1 (Yu et al., 2012), ITPK1 (Zhang et al., 2012), HIPK2 (Shima et al., 2008), AIRE (Incani et al., 2014), SIK2 (Yang et al., 2013), RNAPolII (Schroder et al., 2013), PCNA (Cazzalini et al., 2014), MLL1 (Huang et al., 2018), GCPII (Choi et al., 2014), TAU (Chen et al., 2020a), PLZF (Guidez et al., 2005), KLF4 (Evans et al., 2007), FOXM1 (Lv et al., 2016), CAF1A (Sharma et al., 2016), PYGO2 (Andrews and Kao, 2016), RAD52 (Yasuda et al., 2018), HIC1 (Stankovic-Valentin et al., 2007).
TIP60	ATM (Sun et al., 2005), p53 (Wang et al., 2019a), NOTCH1 (Kim et al., 2007), DNMT1 (Du et al., 2010), SRSF2 (Edmond et al., 2011), ULK1 (Nie et al., 2016), p21 (Lee et al., 2013), SPZ1 (Wang et al., 2019b), TWIST (Wang et al., 2019b), SOX4 (Jang et al., 2015), AURORA-B (Mo et al., 2016), PRAK (Zheng et al., 2013a), SMAD3 (Li et al., 2018), SRSF5 (Chen et al., 2018).
PCAF	p53 (Jin et al., 2002), HMG-I(Y) (Munshi et al., 1998), HMG-17 (Herrera et al., 1999), MyoD (Sartorelli et al., 1999), DEK (Ko et al., 2006), NFE4 (Zhao et al., 2004), PTEN (Okumura et al., 2006), PARP1 (Ota et al., 2003), SATB1 (Pavan Kumar et al., 2006), FLI1 (Asano et al., 2007), USF1 (Huang et al., 2007), BUBR1 (Choi et al., 2009), CyclinA (Mateo et al., 2010), CDK2 (Mateo et al., 2009), ERRα (Wilson et al., 2010), HIF1A (Yoon et al., 2014), pRB (Nguyen et al., 2004), CX43 (Colussi et al., 2011), PKM2 (Wu et al., 2023), eIF5A (Ishfaq et al., 2012), EB1 (Xia et al., 2012), ACLY (Lin et al., 2013), HOXA10 (Zhu et al., 2013), GAPDH (Ventura et al., 2010), ALDH1A1 (Zhao et al., 2014), EZH2 (Wan et al., 2015), LIN28 (Wang et al., 2014a), MPP8 (Sun et al., 2015), AKT1 (Zhang et al., 2015), RhoGDIα (Kuhlmann et al., 2016), CIDEC (Qian et al., 2017), MKL1 (Yu et al., 2017), IRF2 (Masumi et al., 2006).
GCN5	PPARGC1A (Kumar and Puthussery, 2025), CDC6 (Paolinelli et al., 2009), PPARGC1B (Kelly et al., 2009), CDK5 (Lee et al., 2014), CAVIN1 (Zhou et al., 2017), EGR2 (Wang et al., 2017b).
KAT9	G6PD (Wang et al., 2014b), PGK1 (Wang et al., 2015a).
KAT7	GαS (Zhou et al., 2023).
MOF	CCAR2 (Zheng et al., 2013b),KDM1A (Luo et al., 2016).
ARD1	HIF1A (Jeong et al., 2002), NAA10 (Kuhns et al., 2018), AR (Jaiswal et al., 2022), AURKA (Vo et al., 2017), SAMHD1 (Lee et al., 2017).
HAT1	PLZF (Sadler et al., 2015).
MOZ	p53 (Rokudai et al., 2013).
ESCO1	SMC3 (Minamino et al., 2015).

In summary, protein acetylation is a versatile and essential regulatory mechanism governing fundamental cellular processes. Its dynamic balance, maintained by KATs and deacetylases, is critical for cellular homeostasis, and its disruption drives the pathogenesis of various diseases. Further exploration of acetylation mechanisms and targeted modulation of acetylation-related enzymes hold great potential for advancing disease diagnosis and treatment.

## 3 The role of protein acetylation in the malignant progression of gliomas

**FIGURE 2:**
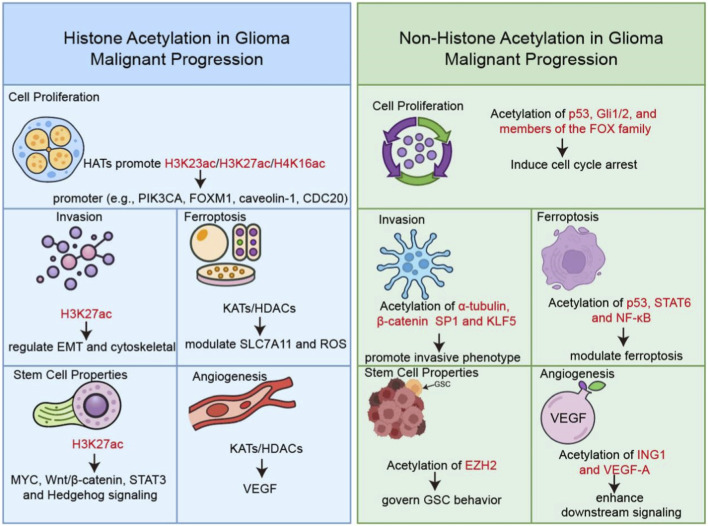
Histone and non-histone acetylation in glioma malignant progression.

Acetylation modification exerts a pivotal regulatory role in multiple biological processes closely associated with glioma malignant progression, including cell proliferation, migration, invasion, angiogenesis, and immunosuppressive microenvironment formation. Its dysregulation drives the aggressive phenotype of gliomas through precise modulation of histone and non-histone protein functions (Chen R. et al., 2020; Williams et al., 2017). As one of the most well-characterized epigenetic modifications, acetylation dynamically balances the activities of histone acetyltransferases (HATs) and histone deacetylases (HDACs), which catalyze the addition and removal of acetyl groups from lysine residues, respectively (Kouzarides, 2007). Notably, acetylation does not function independently but engages in extensive crosstalk with other post-translational and epigenetic modifications, including methylation, ubiquitination, and phosphorylation. Such crosstalk occurs at the levels of histone cross-regulation, non-histone protein co-modification, and enzymatic interplay between writers and erasers of different modifications, forming a multilayered epigenetic network that governs glioma malignancy. Beyond histones, acetylation also targets a wide array of non-histone proteins, including transcription factors, enzymes, and cytoskeletal components, thereby integrating into diverse signaling networks that govern glioma biology (Dancy and Cole, 2015). In this section, we comprehensively dissect the multifaceted roles of acetylation in glioma malignant progression, highlighting molecular mechanisms and clinical implications (Figure 2).

### 3.1 Acetylation regulates glioma cell proliferation

Uncontrolled proliferation is a hallmark of glioma malignancy, and histone acetylation exerts a vital role in regulating this process by transcriptionally controlling cell cycle-related genes. For instance, KAT6A catalyzes H3K23 acetylation at the PIK3CA promoter, recruits the nuclear receptor binding protein TRIM24, and activates PI3K/AKT signaling, thereby promoting glioma cell proliferation, colony formation, and tumor growth (Lv et al., 2017). HMGCL enhances intracellular acetyl-CoA level, promotes H3K27ac enrichment, activates FOXM1 transcription, and stimulates β-catenin signaling, thereby sustaining glioma stemness and tumor growth while pharmacological inhibition of HMGCL suppresses GBM cell proliferation (Sun Y. et al., 2024). Similarly, FABP7 regulates nuclear acetyl-CoA levels through interaction with caveolin-1, leading to increased H3K27ac at the caveolin-1 promoter and accelerated glioma cell proliferation (Kagawa et al., 2022). Additionally, chromatin-associated proteins such as HMGN2 promote glioma cell proliferation by stabilizing H3K27ac at promoters of key mitotic genes, including CDC20 (Zhong et al., 2025). Under hypoxic conditions, upregulation of HAT1 facilitates GBM cell proliferation *via* acetylation-dependent stabilization of HIF2α and sustained activation of hypoxia-responsive genes such as VEGFA (Kumar et al., 2023). Notably, histone acetylation does not uniformly promote cell proliferation, and H4K16ac exerts an inhibitory effect on glioma cell proliferation and is dynamically repressed by oncogenic KRas–ERK1/2 signaling through coordinated regulation of SIRT2 and TIP60 (Wei et al., 2019). Collectively, these findings underscore the dual roles of histone acetylation in regulating glioma proliferation.

Non-histone acetylation has increasingly been recognized as a pivotal epigenetic mechanism governing glioma cell proliferation and malignant progression by finely modulating the functional state of numerous oncogenic and tumor-suppressive proteins. Accumulating evidence demonstrates that acetylation of transcription factors, including p53, Gli1/2, and members of the FOX family, profoundly influences their transcriptional activity, DNA-binding affinity, and protein stability, thereby precisely controlling cell-cycle progression, cell survival, and proliferative capacity (Gong et al., 2025; Canettieri et al., 2010). In gliomas, dysregulated acetylation can attenuate tumor-suppressive signaling, for example, Smad1 competitively disrupts the p300–p53 interaction, reduces p53 acetylation, and consequently promotes tumor growth and therapeutic resistance (Gong et al., 2025). Conversely, aberrant non-histone acetylation can potentiate multiple oncogenic pathways through acetylation-dependent activation of key signaling molecules such as Akt1, β-catenin and HIF2A, sustaining proliferative signaling under diverse stress conditions (Zhang et al., 2015). Beyond transcriptional regulation, non-histone acetylation also plays an essential role in metabolic reprogramming. Acetylation of metabolic enzymes, including HMGCS1 and PFKP, tightly couple acetyl-CoA metabolism and glycolytic flux to oncogenic signaling networks, thereby supporting the high metabolic demand and rapid proliferation of glioma cells (Lee et al., 2018; Zhao L. et al., 2024). Collectively, non-histone acetylation acts as a central integrator linking transcriptional control, signal transduction, and metabolic adaptation, highlighting its critical contribution to glioma proliferation and underscoring its potential as a promising therapeutic target for precision intervention.

### 3.2 Acetylation promotes glioma cell invasion and migration

Diffuse invasion into the surrounding normal brain tissue is a defining feature of GBM that renders complete surgical resection impossible, and acetylation modification plays a critical role in driving this invasive phenotype by regulating EMT, cytoskeletal remodeling, and matrix metalloproteinase (MMP) expression. Histone acetylation plays a fundamental role in shaping the invasive and infiltrative behavior of gliomas by reprogramming chromatin structure and transcriptional networks that govern cell motility, extracellular matrix remodeling, and metabolic adaptation. Multiple studies demonstrate that aberrant histone acetylation at specific genomic loci activates invasion-associated gene programs in glioma cells. For instance, the long non-coding RNA CRNDE is upregulated in a histone acetylation–dependent manner at its promoter, and its overexpression significantly enhances glioma cell migration. Notably, CRNDE expression is further modulated by the mTOR signaling pathway, forming an epigenetic–signaling regulatory network that drives glioma invasiveness (Wang Y. et al., 2015). Similarly, the antisense RNA AS-IL6 promotes glioblastoma invasion by inducing H3K27ac enrichment at the IL6 promoter, thereby activating the IL-6/STAT3 axis; depletion of AS-IL6 suppresses the invasive phenotype, which can be rescued by exogenous IL-6 (Wang et al., 2016). In addition, HMGA2 cooperates with the histone acetyltransferase GCN5 to induce chromatin conformational remodeling at the MMP2 promoter, epigenetically activating its transcription and facilitating extracellular matrix degradation and invasion (Zhang S. et al., 2018). Moreover, inhibition of HDAC3 elevates H3K27ac levels and promotes BRD4 recruitment to the GLI1 promoter, activating the GLI1/IL6/STAT3 pathway and enhancing the malignant potential of glioma stem cells. Importantly, BRD4 inhibitors can block this pathway and synergize with HDAC3 inhibition to suppress glioma cell invasion (Wang Q. et al., 2020).

Non-histone acetylation regulates glioma migration and invasion by modulating protein stability, subcellular localization, signaling activity, and cytoskeletal dynamics. A central mechanism involves the acetylation status of α-tubulin, which critically determines microtubule stability and cell motility. HTRA1 promotes glioma migration by facilitating the interaction between HDAC6 and α-tubulin, thereby reducing α-tubulin acetylation and enhancing cytoskeletal remodeling; conversely, HTRA1 knockdown suppresses invasion and induces apoptosis (Zhao W. et al., 2024). In contrast, FSD1 acts as an invasion suppressor by binding to HDAC6 and inhibiting its deacetylase activity, maintaining α-tubulin acetylation and preventing microtubule depolymerization. However, phosphorylation of FSD1 by CDK5 disrupts this inhibitory interaction, promoting an invasive phenotype (Xiao et al., 2025). Beyond cytoskeletal regulation, non-histone acetylation also controls oncogenic signaling pathways. The histone acetyltransferase Tip60, functioning as a tumor suppressor in glioma, inhibits NF-κB–dependent MT1-MMP transcription, thereby restraining cell adhesion and invasion (Takino et al., 2016). Conversely, ZNF326 transcriptionally activates HDAC7, which reduces β-catenin acetylation at K49, promotes its nuclear translocation, and enhances Wnt signaling–driven invasion (Yu et al., 2020). Additionally, HDAC4 deacetylates transcription factors SP1 and KLF5, synergistically upregulating MKK7 transcription and activating the JNK/c-Jun pathway to drive glioma malignancy. Pharmacological or genetic inhibition of HDAC4 effectively reverses these invasive phenotypes, highlighting acetylation-modulating enzymes as promising therapeutic targets to restrain glioma infiltration and progression (Wang Y. et al., 2019).

Together, histone and non-histone acetylation form an integrated regulatory axis that governs glioma migration and invasion at multiple levels—from chromatin remodeling and transcriptional control to cytoskeletal organization and signal transduction—underscoring acetylation-modifying enzymes as attractive therapeutic targets to limit glioma infiltration and recurrence.

### 3.3 Acetylation modulates glioma cell ferroptosis

As a pivotal epigenetic modification, acetylation influences ferroptosis in glioma cells primarily by modulating antioxidant defense mechanisms. Histone deacetylases (HDACs) and histone acetyltransferases (HATs) work synergistically to maintain the balance of histone acetylation, and the dysregulation of this balance is closely associated with the occurrence and progression of glioma (Neganova et al., 2022). HDACi can elevate acetylation levels by suppressing HDAC activity, and this acetylation elevation further interferes with ferroptosis-related signaling pathways, ultimately inducing ferroptosis in glioma cells—an effect that has been validated by core ferroptosis readouts in glioma-specific models (Parveen et al., 2023).

The cystine/glutamate antiporter SLC7A11 (also known as xCT) is a core molecule in ferroptosis regulation; it promotes glutathione (GSH) synthesis by mediating cystine influx, enhances cellular antioxidant capacity to inhibit ferroptosis, and is abnormally overexpressed in glioma (Koppula et al., 2021). Studies have demonstrated that HDACi such as SAHA (vorinostat) can specifically downregulate the expression of SLC7A11 in glioma cells, reduce GSH production, increase reactive oxygen species (ROS) levels, and ultimately induce ferroptosis (Wolf et al., 2014). In addition, SIRT3, a class III HDAC, enhances the activity of antioxidant enzymes through deacetylation modification to maintain intracellular redox homeostasis. Its dysfunction impairs the resistance of glioma cells to ferroptosis, providing a potential target for HDACi intervention (Arrizabalaga et al., 2017). Notably, the regulatory role of acetylation in antioxidant defense is not limited to direct enzyme modification but also involves the transcriptional regulation of key antioxidant genes, forming a complex regulatory network in glioma ferroptosis.

Acetylation also participates in the ferroptosis process of glioma by regulating the activity of transcription factors, among which the acetylation modification of p53 protein plays a central role. As a classic tumor suppressor, p53’s acetylation level directly affects the transcriptional efficiency of ferroptosis-related genes. It can promote ferroptosis in glioma cells and inhibit tumor growth by downregulating SLC7A11 expression (Hashizume et al., 2014). HDACi such as MPT0B291 can effectively suppress glioma proliferation both *in vitro* and *in vivo* by increasing p53 acetylation levels (Buyandelger et al., 2020). Accumulating evidence indicates that the acetylation of p53 not only enhances its transcriptional activity but also modulates its target gene selectivity, making it a key switch in regulating ferroptosis. Meanwhile, the acetylation regulation of transcription factors such as STAT6 and NF-κB also participates in the ferroptosis regulatory network: STAT6 can competitively bind to the acetyltransferase CREB-binding protein, thereby regulating the p53/SLC7A11 pathway to inhibit ferroptosis (Yang et al., 2022); in contrast, the activation of the NF-κB pathway can upregulate the expression of the iron-sequestering cytokine LCN2, reducing the sensitivity of glioma cells to ferroptosis inducers (Lan et al., 2023). These findings suggest that acetylation modulates ferroptosis in glioma through multiple transcription factor-mediated pathways, laying a theoretical foundation for the development of targeted therapies.

In summary, acetylation, as a key epigenetic modification, regulates ferroptosis in glioma cells through two core pathways: modulating antioxidant defense mechanisms and regulating transcription factor activity—both supported by core ferroptosis validation criteria (iron dependency, lipid peroxidation, rescue by ferroptosis inhibitors) and clear distinction between direct glioma evidence and extrapolated mechanistic insights. The balance of histone acetylation maintained by HDACs and HATs is crucial for glioma progression, and HDACi exert anti-tumor effects by elevating acetylation levels to interfere with ferroptosis-related networks. Specifically, HDACi such as SAHA and MPT0B291 target SLC7A11 and p53 respectively, inducing ferroptosis by disrupting redox homeostasis and regulating gene transcription, with all effects validated by ferroptosis-specific readouts (MDA, 4-HNE, C11-BODIPY, and rescue experiments). Transcription factors including p53, STAT6, and NF-κB further refine this regulation by their acetylation status, forming a complex regulatory network. These insights highlight the potential of acetylation-targeted strategies, especially HDACi, in glioma therapy. Future research should focus on optimizing HDACi specificity and delivery efficiency to translate these mechanisms into effective clinical interventions for glioma.

### 3.4 Acetylation maintains glioma stem cell properties

Glioma stem cells (GSCs), a subpopulation of tumor cells with self-renewal capacity and pluripotency, are responsible for tumor initiation, progression, recurrence, and therapeutic resistance. Acetylation modification plays a central role in maintaining GSC properties by cooperating or antagonizing other epigenetic modifications, particularly histone methylation and ubiquitination, to orchestrate stemness-associated transcriptional programs.

Histone acetylation at promoters and enhancers is closely linked to the activation of stemness-associated genes in GSCs. Elevated levels of active acetylation marks, such as H3K27ac, characterize GSC-specific enhancers and super-enhancers that regulate oncogenic transcriptional networks, including MYC, Wnt/β-catenin, STAT3 and Hedgehog signaling (Va et al., 2020; Monje et al., 2011; Zhou et al., 2018). Mechanistically, acetylation and methylation often exert mutually antagonistic functions at the same chromatin loci: for instance, H3K27ac and H3K27me3 act as opposing epigenetic marks, whose dynamic balance dictates the switch between GSC self-renewal and differentiation. These acetylation-enriched regulatory elements facilitate the persistent expression of genes required for GSC maintenance while suppressing differentiation-associated function. In H3K27M-mutant pediatric GSCs, aberrant H3K27ac enrichment forms heterotopic nucleosomes, driving oncogene expression and tumor progression, which can be targeted by bromodomain (BRD) inhibitors (Piunti et al., 2017).

Non-histone acetylation also governs GSC behavior *via* extensive crosstalk with methylation and ubiquitination. EZH2, a key Polycomb protein with histone methyltransferase activity, is acetylated to enhance its methylation of STAT3, reinforcing GSC self-renewal (Va et al., 2020). This represents a paradigmatic synergistic crosstalk: acetylation stabilizes and activates a methyltransferase, which in turn reinforces repressive histone methylation to sustain stemness. Conversely, acetylation of transcription factors and signaling molecules often counteracts ubiquitin-dependent proteasomal degradation, thereby prolonging the half-life of core stemness regulators. HDAC inhibitors (HDACis) disrupt GSC properties by inducing acetylation-dependent differentiation, cell cycle arrest and apoptosis. Valproic acid (VPA), a classic HDACi, modulates GSC DNA methylation, activates Wnt/β-catenin signaling, and reduces invasiveness, though its synergism with temozolomide remains controversial (Riva et al., 2016; Riva et al., 2018). Notably, acetylation crosstalks with other epigenetic pathways in a highly synergistic manner in GSCs. HDACi-induced global acetylation synergizes with KDM1A inhibition to suppress GSC viability (Singh et al., 2011; Singh et al., 2015). Similarly, the combinatorial targeting of acetylation regulators (HDACs/BRDs) and methylation modifiers (EZH2/KDMs) produces stronger anti-GSC effects than single-agent treatment, highlighting the functional interdependence between acetylation and methylation in maintaining glioma stemness. Dysregulated acetylation also mediates GSC microenvironment adaptation, contributing to therapeutic resistance. Targeting acetylation networks, either alone or in combination, emerges as a promising strategy to eradicate GSCs and improve glioblastoma outcomes. Collectively, these studies establish histone acetylation as a fundamental epigenetic mechanism sustaining glioma stem cell properties *via* dynamic crosstalk with histone methylation, ubiquitination, and other post-translational modifications, and highlight acetylation-centered regulatory networks as promising therapeutic vulnerabilities in GBM.

### 3.5 Acetylation mediates glioma angiogenesis

Angiogenesis is a core driver of glioma malignant progression, directly affecting tumor nutrient supply, invasiveness, and patient prognosis (Plate et al., 2012). Recent studies have shown that acetylation, as a key epigenetic modification, regulates glioma angiogenesis through multiple pathways, including modulating histone and non-histone targets, mediating endothelial progenitor cell (EPC) functions, and linking inflammatory signaling, thereby providing novel epigenetic targets for anti-angiogenic therapy (Luo et al., 2025). In glioma cell lines LN229 and T98G, 5-Aza-2′-deoxycytidine (5-Aza-dC) increases histone H3 acetylation and H3K4me2 levels, inducing heparanase expression, which play a critical role in glioma angiogenesis (Hong et al., 2012). Additionally, TSA can induce the expression of the major ING1 isoform p33, a type II tumor suppressor involved in histone modification and DNA damage response, and ING1 has been shown to participate in glioma-induced angiogenesis, serving as a cross-node that links acetylation-regulated angiogenesis to apoptosis through the FADD/caspase 3 signaling pathway (Tamannai et al., 2010).

EPCs, critical initiators of tumor neovascularization, are recruited by the glioma microenvironment to differentiate into mature endothelial cells. CD34/CD133 cells isolated from human umbilical cord blood can be induced into functional EPCs, capable of uptaking ac-LDL, binding lectins, expressing VEGFR-2, CD31 and vWF, forming capillary-like structures, which accounted for 18% ECs of the tumor microvessels. VEGF-A recruits bone marrow-derived EPCs to glioma stroma, and acetylation may regulate EPC recruitment and function *via* the VEGF pathway (Zhang et al., 2009). Collectively, accumulating evidence indicates that acetylation plays a multifaceted role in regulating glioma angiogenesis by coordinating epigenetic control of tumor cells, endothelial progenitor cell recruitment, and inflammatory signaling pathways. Through modulation of both histone and non-histone substrates, acetylation influences the expression of angiogenesis-related genes, endothelial differentiation, and tumor–stroma interactions. Histone deacetylase inhibitors exhibit robust anti-angiogenic effects in preclinical glioma models, even in genetically aggressive contexts, highlighting their therapeutic potential. However, the complexity of acetylation-mediated regulation underscores the need for deeper mechanistic understanding to rationally design anti-angiogenic strategies targeting acetylation regulation. Notably, the diverse oncogenic functions of protein acetylation in driving glioma cell proliferation, invasion, ferroptosis, stem cell properties and angiogenesis also establish a molecular basis for therapy resistance, which represents a major obstacle in clinical management. These findings highlight the critical relevance of acetylation regulation as a promising target for overcoming therapeutic resistance and improving glioma treatment outcomes.

## 4 The status of acetylation in glioma therapy

Despite advances in multimodal therapy, most glioma patients develop therapeutic resistance, which is the primary cause of treatment failure and poor prognosis. Acetylation modification plays a pivotal role in mediating resistance to chemotherapy, radiotherapy, and targeted therapy by regulating DNA damage repair, cell cycle checkpoints, cancer stemness, and immune evasion.

### 4.1 Acetylation-mediated resistance to chemotherapy

Temozolomide (TMZ) is the first-line chemotherapeutic agent for glioblastoma (GBM), the most aggressive primary brain tumor, yet its clinical efficacy is severely compromised by the emergence of acquired resistance. The primary mechanism underlying TMZ resistance is the expression of O6-methylguanine-DNA methyltransferase (MGMT), an enzyme that directly repairs TMZ-induced DNA alkylation damage to maintain genomic stability (Shaw et al., 2024). However, accumulating evidence indicates that acetylation modification, a key epigenetic regulatory mechanism, also plays a critical and multifaceted role in TMZ resistance by modulating DNA damage repair pathways, cancer stem cell properties and gene transcription (Wei et al., 2021). Histone acetylation, in particular, serves as a pivotal regulator of MGMT expression through facilitating the recruitment of specific transcription factors. For instance, elevated levels of histone H3 lysine 9 acetylation (H3K9ac) enhance the binding of transcription factor SP1 to the MGMT gene locus, thereby upregulating MGMT transcription and driving TMZ resistance (Ye et al., 2024). Additionally, Fstl1, a glycoprotein highly expressed in GBM, promotes temozolomide resistance in glioblastoma by increasing H3K9 acetylation at the MGMT promoter, enhancing MGMT transcription through disruption of the DIP2A–HDAC2 complex (Nie et al., 2019).

Key histone acetyltransferases (HATs) and their associated complexes are pivotal regulators of TMZ resistance in GBM. The P300/CBP HAT family, in particular, is closely linked to glioma stem cell (GSC) phenotypic plasticity and therapy resistance, which are major drivers of GBM recurrence. P300 forms a functional complex with RBBP4 to regulate the expression of pro-survival genes such as c-MYC, and disrupting this complex with the small-molecule inhibitor CPI-1612 effectively reduces histone acetylation levels, impairs pro-survival signaling, and sensitizes GBM cells to TMZ (Mladek et al., 2022). Moreover, the long noncoding RNA Linc00942 interacts with glycolytic enzymes TPI1 and PKM2 to promote their phosphorylation, dimerization, and nuclear translocation. This interaction activates the STAT3/P300 signaling axis, increases histone H3K4 acetylation, and transcriptionally upregulates SOX9—a key regulator of stemness—thereby enhancing both TMZ resistance and the self-renewal capacity of GBM cells (Yang et al., 2024).

The acetylation balance of non-histone proteins also contributes significantly to TMZ resistance in GBM. Smad1 acts as a negative regulator of p53 acetylation by forming a ternary complex with p53 and p300, which inhibits the interaction between p300 and p53, reduces p53 acetylation, and Enhances Smad1 self-acetylation at K373, an essential modification for its oncogenic function, thereby promoting tumor growth and chemoresistance (Gong et al., 2025). Furthermore, Aberrant acetylation regulates TMZ resistance in glioblastoma by modulating DNA damage repair pathways, where HDAC-mediated Sp1 deacetylation promotes PCNA recruitment and attenuates TMZ-induced DNA damage in MGMT-deficient cells (Yang et al., 2019). Furthermore, targeting acetylation-related pathways, such as inhibiting the Smad1-P300 interaction or regulating the SIRT3-SOD2 acetylation axis, emerges as promising therapeutic strategies to overcome TMZ resistance and improve the poor prognosis of GBM patients (Cheng et al., 2017). Collectively, acetylation critically drives glioblastoma therapeutic resistance by regulating MGMT expression, DNA damage repair, cancer stemness, and pro-survival signaling. Targeting acetylation writers, erasers, and readers offers a promising strategy to overcome temozolomide resistance and improve treatment efficacy.

### 4.2 Acetylation in radiotherapy resistance

Radiotherapy remains a cornerstone in the treatment of malignant brain tumors. However, radioresistance significantly limits its clinical efficacy and contributes to tumor recurrence. Emerging evidence highlights dysregulated acetylation therefore represents a key epigenetic mechanism underlying radioresistant phenotypes in malignant brain tumors (Wu et al., 2026).

Histone acetylation directly influences chromatin structure and transcriptional function following radiation exposure, thereby shaping radioresistance. In GBM, H3K27ac exhibits context-dependent roles in radiation responses. In radioresistant GSCs, IR enhances H3K27ac enrichment at the CD47 promoter through HDAC7-dependent mechanisms, leading to increased CD47 expression and suppression of macrophage-mediated phagocytosis, thereby promoting immune evasion after radiotherapy (Sun et al., 2024b). Moreover, pharmacological inhibition of bromodomain and extraterminal (BET) proteins disrupts H3K27ac-marked enhancers at DNA damage repair genes, impairing repair capacity and sensitizing DMG cells to radiation. These findings underscore the dual and highly contextual roles of histone acetylation in regulating both intrinsic radioresistance and post-radiotherapy immune escape (Watanabe et al., 2024).

Beyond chromatin regulation, non-histone protein acetylation plays a crucial role in orchestrating radioresistant signaling networks. Acetylation of ANXA2 at lysine 10 enhances its interaction with USP4, reinforcing STAT3-driven survival signaling in GSCs (Tu et al., 2025). Similarly, spermidine/spermine N^1^-acetyltransferase 1 (SAT1), a polyamine catabolic enzyme frequently upregulated in GBM, promotes global histone H3 acetylation to transcriptionally activate BRCA1, thereby enhancing homologous recombination repair and resistance to IR. Depletion of SAT1 reduces BRCA1 expression and sensitizes GBM cells to radiotherapy (Brett-Morris et al., 2014). Acetylation-dependent regulation of inflammatory signaling also contributes to radioresistance: acetylated NF-κB p65/RelA is recognized by BRD4, driving inducible nitric oxide synthase (iNOS) expression in photostressed GBM cells. Elevated nitric oxide production promotes post-irradiation proliferation and invasion, a process that can be effectively suppressed by BET inhibitors (Fahey et al., 2018). In addition, targeting acetylation-related pathways represents a promising strategy to overcome radiotherapy resistance. Histone deacetylase inhibitors (HDACis), such as PXD101, enhance histone H3 and H4 acetylation, induce GADD45A expression, activate the p38 signaling pathway, and promote radiation-induced apoptosis in GBM cells (Sharma et al., 2023). Similarly, diallyl trisulfide (DATS), a naturally derived HDACi, increases histone acetylation, suppresses tumor angiogenesis, and significantly enhances radiotherapy efficacy in preclinical GBM models without inducing hepatotoxicity (Das et al., 2018).

Collectively, these studies identify acetylation as a central epigenetic hub that integrates chromatin remodeling, signal transduction, and tumor–immune interactions to drive radioresistance. Therapeutic strategies targeting acetylation-dependent regulatory networks, either alone or in combination with radiotherapy, represent a rational and promising approach to enhance treatment responses and improve clinical outcomes in malignant brain tumors.

### 4.3 Targeting acetylation for glioma therapy

Given the critical role of acetylation modification in glioma malignant progression and therapeutic resistance, targeting KATs and HDACs has emerged as a promising therapeutic strategy. A variety of KAT and HDAC inhibitors have been developed, and many are currently under preclinical and clinical evaluation for the treatment of gliomas (Pathikonda et al., 2024) (Table 2).

**TABLE 2:** Acetylation-related inhibitors for glioma treatment.

Class	Drug	Target	Phase	Efficacy
HAT inhibitors	HATi II (Xu et al., 2014)	P300/CBP	Pre-clinical	Induce cell-cycle arrest
	C646 (Tao et al., 2020)	P300/CBP	Pre-clinical	Trigger caspase-dependent apoptosis
	CPI-1612 (Mladek et al., 2022)	P300/CBP	Pre-clinical	Favorable brain penetrance
	cpd.618 (Gong et al., 2025)	p300–Smad1	Pre-clinical	Restoring p53 acetylation
BET inhibitors	JQ1 (Moujalled et al., 2022)	Bromodomain	Pre-clinical	Induce cell cycle arrest and apoptosis
	OTX015 (Berenguer-Daize et al., 2016)	Bromodomain	IIa	Combination with temozolomide (TMZ) produces additive antiproliferative effects
	I-BET151 (Pastori et al., 2014)	Bromodomain	Pre-clinical	Inhibit cell proliferation and neurosphere formation
	Trotabresib (Moreno et al., 2023)	Bromodomain	Ib	Good BBB penetration and tolerability
Pan-HDAC Inhibitor	vorinostat (Berghauser et al., 2015)	HDAC	II	GBM angiogenesis and tumor growth
	panobinostat (Fouladi et al., 2010)	HDAC	II	Induce autophagy
	Entinostat (Bangert et al., 2012)	HDAC	II	Promote cell apoptosis
	Trichostatin A (Tamannai et al., 2010)	HDAC	N/A	Induce p53/p21-mediated cell-cycle arrest
	Givinostat (Angeletti et al., 2016)	HDAC	Pre-clinical	Inhibit GSC proliferation
	Tinostamustine (Festuccia et al., 2018)	HDAC	I	Enhance radiosensitivity
	Scriptaid (Sharma et al., 2010)	HDAC	Pre-clinical	Trigger JNK-dependent apoptosis
	Valproic acid (Shu et al., 2006)	HDAC	IV	Reduce invasiveness
Subtype-selective HDAC inhibitor	Pyroxamide (Zhang et al., 2021)	HDAC1	Pre-clinical	Suppress EMT
	RGFP109 (Cheng et al., 2023)	HDAC1/3	Pre-clinical	Suppress EMT
	Romidepsin (Bahia et al., 2023)	HDAC2	II	Inhibit GBM cell proliferation
	RGFP966 (Liang et al., 2020)	HDAC3	Pre-clinical	Suppress GSC proliferation and self-renewal
	MPT0B291 (Buyandelger et al., 2020)	HDAC6	Pre-clinical	Inhibit GBM cell proliferation
	Sahaquines (Zhang et al., 2018b)	HDAC6	Pre-clinical	Reduce GBM cell viability
	J22352 (Liu et al., 2019)	HDAC6	Pre-clinical	Suppresses GBM malignant phenotypes
	JOC1 (Auzmendi-Iriarte et al., 2020)	HDAC6	Pre-clinical	Inhibit cell proliferation, migration, colony formation and EMT
	hydroxamic acid 16 (Nepali et al., 2021)	HDAC6	Pre-clinical	Inhibit cell proliferation, migration, colony formation and EMT
	CAY10603 (Nepali et al., 2021)	HDAC6	Pre-clinical	Inhibit cell proliferation, migration, colony formation and EMT
​	F0911-7667 (Yao et al., 2018)	Sirt1	Pre-clinical	Induce autophagic cell death
​	SRT2183 (Ye et al., 2019)	Sirt1	Pre-clinical	Induce endoplasmic reticulum stress
	EX527 (Wang et al., 2020b)	Sirt1	I	Trigger GBM cell apoptosis
	AGK2 (Funato et al., 2018)	Sirt2	Pre-clinical	Suppress the malignant phenotypes of GBM cells
	Compound 18 (Schnekenburger et al., 2017)	Sirt1/Sirt2	Pre-clinical	Inhibit GBM cell growth

#### 4.3.1 HAT inhibitors

Histone acetyltransferases (HATs) play critical regulatory roles in glioma initiation and progression, with the p300/CBP complex representing one of the most extensively characterized acetyltransferases in this context (He et al., 2021). Aberrant activation of p300/CBP promotes glioma malignancy through multiple mechanisms, including chromatin remodeling and transcriptional activation of oncogenic programs. p300/CBP forms functional complexes with proteins such as SATB2, REST and RBBP4 to induce the expression of oncogenes including FOXM1, KIF15 and C-MYC, thereby sustaining glioma cell proliferation and maintaining glioma stem cell (GSC) properties (Mladek et al., 2022; Yu et al., 2024; Tao et al., 2020). In addition, p300/CBP can be recruited by Smad1, which interferes with p300-mediated p53 acetylation, resulting in p53 hypoacetylation, attenuation of tumor-suppressive signaling, and enhanced chemoresistance (Gong et al., 2025).

In recent years, increasing attention has been directed toward the development of small-molecule HAT inhibitors as potential therapeutic agents. To date, more than 20 HAT inhibitors have been reported, although their application in glioblastoma remains limited (Pathikonda et al., 2024). Several p300/CBP-selective inhibitors have demonstrated robust anti-glioma activity in preclinical models. For instance, HATi II and C646 effectively suppress glioma cell proliferation, induce G2/M cell-cycle arrest and trigger caspase-dependent apoptosis, accompanied by broad transcriptional reprogramming of p300/CBP-regulated genes (Yu et al., 2024; Tao et al., 2020; Xu et al., 2014). Notably, C646 disrupts SATB2/CBP and REST/p300 signaling axes that are critical for glioma progression. CPI-1612, a next-generation p300/CBP inhibitor with favorable brain penetrance, has shown synergistic effects with temozolomide (TMZ), effectively reversing chemoresistance in glioma models (Mladek et al., 2022).

Moreover, the small molecule cpd.618 specifically disrupts the p300–Smad1 interaction, restoring p53 acetylation and sensitizing glioma cells to chemotherapy, highlighting the feasibility of selectively targeting pathological p300 interactions rather than globally suppressing acetyltransferase activity (Gong et al., 2025). Although clinical translation of HAT inhibitors in glioblastoma is still in its early stages, accumulating evidence underscores their therapeutic potential, either as monotherapies or in rational combination regimens. Further optimization of inhibitor selectivity, brain delivery, and safety profiles will be essential to advance HAT-targeted strategies into clinical application for glioma treatment.

#### 4.3.2 Bromodomain (BET) inhibitors

Bromodomain and extraterminal (BET) family proteins, especially BRD4, are key epigenetic readers of histone acetylation that drive glioma malignant progression by regulating oncogenic transcription, glioblastoma stem cell (GSC) stemness maintenance and DNA damage repair (Duan et al., 2023). BRD4 is overexpressed in glioblastoma (GBM) tissues and cells, and its expression is negatively correlated with GBM prognosis, making it a promising therapeutic target for glioma (Yang et al., 2021). Preclinical studies have confirmed that BET inhibitors exert anti-glioma effects by competitively binding to the bromodomains of BET proteins, displacing them from chromatin and blocking the activation of downstream oncogenic signaling pathways.

JQ1, the first well-characterized BET inhibitor, shows potent anti-GBM activity in preclinical models with excellent blood-brain barrier (BBB) permeability (Moujalled et al., 2022). It induces G1 cell cycle arrest and apoptosis in GBM cells and GSCs by downregulating c-MYC, Bcl-2 and other oncogenes, and upregulating p21 and p27, and significantly prolongs the survival of mice bearing orthotopic GBM xenografts (Cheng et al., 2013). OTX015, another classic BET inhibitor, has higher potency than JQ1 in GBM cell lines, and its combination with temozolomide (TMZ) produces additive antiproliferative effects and prolongs mouse survival without obvious toxicity (Berenguer-Daize et al., 2016). I-BET151 inhibits GBM cell proliferation and neurosphere formation by targeting the Notch1/Hes1 pathway and regulating lncRNAs such as HOTAIR, and effectively suppresses tumor growth in intracranial xenotransplantation models (Pastori et al., 2014).

Combination therapy with BET inhibitors and conventional anti-glioma strategies further enhances therapeutic efficacy and overcomes treatment resistance. BET inhibitors synergize with TMZ by increasing DNA damage and downregulating MGMT expression, and sensitize GBM cells to radiotherapy by inhibiting DNA double-strand break repair (Cancer et al., 2019). JQ1 also improves the efficacy of photodynamic therapy (PDT) by suppressing the NF-κB/BRD4-mediated upregulation of iNOS, and enhances the anti-GBM effect of EGFR CAR-T therapy by reducing the immunosuppressive tumor microenvironment (Fahey et al., 2018). In addition, the combination of BET inhibitors with HDAC inhibitors, mTOR inhibitors or EGFR TKIs shows synergistic antitumor effects by inducing more significant cell cycle arrest and apoptosis (Duan et al., 2023).

Clinical translation of BET inhibitors for glioma is still in the early stage. Trotabresib has shown good BBB penetration and tolerability in phase I/Ib clinical trials for recurrent high-grade glioma, with a 6-month progression-free survival rate of 12% for monotherapy (Moreno et al., 2023). However, the phase II study of OTX015 in recurrent GBM was terminated due to limited efficacy, and JQ1 has not entered clinical trials because of its short half-life (Duan et al., 2023). The development of brain-penetrant BET degraders based on PROTAC technology and the identification of efficacy biomarkers are expected to address the current limitations and promote the clinical application of BET-targeted therapy for glioma. Overall, targeting BET family proteins represents a promising epigenetic strategy for glioma treatment. Advances in brain-penetrant BET inhibitors and degraders, together with rational combination therapies, may overcome current limitations and improve therapeutic responses in glioblastoma patients.

#### 4.3.3 HDAC inhibitors

Histone deacetylases (HDACs) are aberrantly expressed in glioma, with class I HDACs (HDAC1, HDAC2 and HDAC3) markedly upregulated in high-grade gliomas, where they drive malignant progression by activating oncogenic signaling pathways such as PI3K/AKT and MEK/ERK and by sustaining glioma stem cell (GSC) stemness. In contrast, class II and class IV HDACs are generally downregulated in GBM compared with low-grade gliomas (Lucio-Eterovic et al., 2008; Weichert, 2009; Kim, 2014). HDAC inhibitors (HDACi) exert anti-glioma effects by blocking HDAC-mediated histone deacetylation, opening chromatin structure, reactivating silenced tumor suppressor genes, and inhibiting cell cycle progression and angiogenesis (Williams et al., 2017). HDACis represent the most extensively studied class of epigenetic drugs in oncology, with several agents already approved by the U.S. Food and Drug Administration for the treatment of peripheral T-cell lymphoma and multiple myeloma (Cappellacci et al., 2020). Moreover, HDACis can remodel the immunosuppressive tumor microenvironment, reverse temozolomide resistance, and function as radiosensitizers by enhancing DNA damage accumulation in glioma cells (Zang et al., 2018). Importantly, HDACis have shown significant synergistic effects with immunotherapy and metabolic-targeted therapies in preclinical and clinical studies, becoming a core component of combined glioma therapy.

Pre-clinical studies have validated the anti-glioma efficacy of multiple HDACi, including pan-HDACi and subtype-selective inhibitors. Pan-HDAC inhibitors such as vorinostat (SAHA) and panobinostat (LBH589) effectively suppress GBM angiogenesis and tumor growth, in part by inhibiting the AKT–mTOR pathway and disrupting the Hsp90/HDAC6 complex, while also inducing autophagy (Chiao et al., 2013; Yao et al., 2017). Entinostat (MS275), a class I HDAC inhibitor targeting HDAC1, HDAC2, and HDAC3, promotes GBM cell apoptosis through suppression of c-Myc–dependent FLIP expression (Bangert et al., 2012). Trichostatin A (TSA) induces p53/p21-mediated cell-cycle arrest in GBM cells (Sassi Fde et al., 2014). Givinostat (ITF2357) inhibits glioma stem cell (GSC) proliferation but simultaneously activates cytoprotective autophagy, indicating potential benefit from combination with autophagy inhibitors (Angeletti et al., 2016). Tinostamustine, an alkylating HDAC inhibitor, combines DNA-damaging and epigenetic activities to impair DNA repair and enhance radiosensitivity in GBM (Festuccia et al., 2018). In addition, Scriptaid triggers JNK-dependent apoptosis and reduces telomerase activity in glioma cells (Sharma et al., 2010). Collectively, HDAC inhibitors exert anti-glioma effects through apoptosis induction, cell-cycle arrest, angiogenesis suppression, and sensitization to chemo-radiotherapy.

In contrast, Subtype-selective inhibitors show more targeted effects. In glioma/glioblastoma (GBM), HDAC inhibitors targeting different subtypes exert distinct anti-tumor effects with diverse mechanisms: Class I HDAC inhibitors include Pyroxamide (HDAC1) and RGFP109 (HDAC1/3), both suppressing epithelial-mesenchymal transition (EMT) and invasion of GBM cells (Zhang et al., 2021; Cheng et al., 2023). Romidepsin (HDAC2) elevates H3 and H4 acetylation to inhibit the proliferation of brain tumor stem cells (BTSCs) (Bahia et al., 2023) and induces cell cycle arrest in GBM cells with EGFRvIII mutations (Zhao et al., 2020); RGFP966 (HDAC3) upregulates SMAD7 to inhibit the TGF-β signaling pathway, thus suppressing glioma stem cell (GSC) proliferation and self-renewal, though it activates the STAT3/IL-6 pathway and needs combination with other therapies for optimal efficacy (Wang Q. et al., 2020; Liang et al., 2020). Class II HDAC inhibitors are predominantly HDAC6-selective and show potent anti-GBM activity: MPT0B291 disrupts the HDAC6/LINC00461/miR-485-3p/MELK axis to inhibit GBM cell proliferation (Wu AC. et al., 2022); Sahaquines inhibit α-tubulin acetylation, reducing GBM cell viability, invasiveness and tumor recurrence risk (Zhang I. et al., 2018); J22352 suppresses GBM malignant phenotypes and attenuates PD-L1-mediated immunosuppression, holding potential for combination with immunotherapy (Liu et al., 2019); JOC1, hydroxamic acid 16 and CAY10603 all exert significant anti-tumor efficacy in GBM models by inhibiting cell proliferation, migration, colony formation and EMT (Auzmendi-Iriarte et al., 2020; Nepali et al., 2021). For Class III HDACs (Sirtuin family), which exhibit dual roles in GBM, related modulators include activators and inhibitors: Sirt1 activators F0911-7667 and SRT2183 induce autophagic cell death and endoplasmic reticulum stress in GBM cells respectively (Yao et al., 2018; Ye et al., 2019); the Sirt1-specific inhibitor EX527 activates the p53 pathway to trigger GBM cell apoptosis (Wang T. et al., 2020); the Sirt2-specific inhibitor AGK2 suppresses the malignant phenotypes of GBM cells (Funato et al., 2018); Compound 18, a dual Sirt1/Sirt2 inhibitor, effectively inhibits GBM cell growth by targeting both subtypes (Schnekenburger et al., 2017). Notably, most Subtype-selective inhibitors have only been validated in preclinical studies/GBM models and require further research for clinical translation, and no specific inhibitors for HDAC7, HDAC9 and Class IV HDAC11 in GBM have been developed to date. HDAC inhibitors (HDACi) are well recognized for their capacity to remodel the immunosuppressive tumor microenvironment of gliomas, rendering them ideal partners for immunotherapeutic interventions. Notably, HDACi exhibit promising efficacy in combination therapies for glioblastoma (GBM) and other malignant tumors. Common HDACi employed in such combinations include SAHA, panobinostat, Scriptaid, and LBH589 (Berghauser et al., 2015; Wu Y. et al., 2022; Sun et al., 2024c). These agents synergize with immunotherapies (e.g., anti-PD-L1/PD-1 blockade) and oncolytic viruses (oHSV, Delta24-RGD) by regulating regulatory T cell (Treg) activity, enhancing viral propagation, modulating integrin expression levels, and activating multiple cell death pathways—ultimately improving anti-tumor efficacy while exerting limited toxicity on normal cells. Clinical trials of HDACi for glioma are mainly in Phase I/II, with combined therapy showing more promising outcomes than monotherapy. Monotherapy with vorinostat has modest activity in recurrent GBM, with only 15% of patients achieving 6-month progression-free survival (PFS), but prolonged disease stability in a subset of cases (Galanis et al., 2009; Friday et al., 2012). For newly diagnosed GBM, vorinostat combined with TMZ and radiotherapy improves median time to progression to 8.05 months (Brennan et al., 2013). VPA combined with the Stupp regimen significantly extends the median overall survival (OS) of GBM patients to 29.6 months, far exceeding the historical control of 8.6–9.3 months (Krauze et al., 2015). In pediatric diffuse intrinsic pontine glioma (DIPG), vorinostat combined with 13-cis retinoic acid achieves prolonged stable disease, and VPA shows partial responses in refractory pediatric CNS tumors (Fouladi et al., 2010; Su et al., 2011). Panobinostat combined with fractionated stereotactic re-irradiation elevates the 6-month PFS of recurrent HGG to 83%, significantly better than its combination with bevacizumab (30.4%) (Lee et al., 2015; Shi et al., 2016).

Despite promising progress, HDACi-based glioma therapy faces critical challenges. Most HDACi (e.g., panobinostat, vorinostat) are substrates of efflux transporters at the blood-brain barrier (BBB), leading to poor intracranial penetration (Veringa et al., 2013). Non-specific HDAC inhibition causes off-target toxicities (e.g., myelosuppression, thrombocytopenia), and intratumoral genetic heterogeneity limits therapeutic efficacy (Williams et al., 2017). Future directions focus on developing subtype-selective HDACi with enhanced BBB penetration, combining HDACi with epigenetic drugs (e.g., EZH2 inhibitors) or immunotherapy, and implementing personalized therapy based on glioma HDAC expression profiles and molecular subtypes (Zhang et al., 2025; Zang et al., 2018).

In summary, HDAC inhibitors emerge as promising epigenetic agents for glioma therapy, with pan-HDACi and subtype-selective inhibitors exerting anti-tumor effects *via* multiple mechanisms. Combined regimens outperform monotherapies in clinical trials, yet BBB penetration, off-target toxicities and intratumoral heterogeneity remain key hurdles. Future research will prioritize BBB-permeable subtype-selective inhibitors and personalized combinatorial strategies to boost efficacy and minimize side effects.

Collectively, HAT inhibitors, BET inhibitors, and HDAC inhibitors represent three complementary classes of acetylation-targeted agents with distinct mechanisms and therapeutic potentials in glioma. HDAC inhibitors are the most clinically advanced, with demonstrated preclinical and clinical efficacy as monotherapies or combinatorial partners, yet their applications are largely hindered by limited brain penetrance, off-target toxicities, and isoform non-selectivity. BET inhibitors act as epigenetic readers to block oncogenic transcription programs and show potent activity against glioma stem cells, although clinical outcomes have been modest so far, necessitating improved brain-penetrant compounds and rational combination regimens. HAT inhibitors exhibit high specificity toward oncogenic acetyltransferase complexes such as p300/CBP and can reverse chemoresistance by restoring p53 acetylation, but their clinical translation is still in the early stages due to challenges in pharmacological optimization. Together, these strategies highlight the critical value of targeting acetylation in glioma therapy. Future advances will rely on the development of brain-accessible, isoform-selective compounds, predictive biomarkers, and personalized combinatorial approaches to fully exploit the therapeutic potential of acetylation modulation for glioma patients.

## 5 Summary and prospects

Protein acetylation has emerged as a central and highly versatile epigenetic modification that profoundly shapes glioma malignant progression and therapeutic resistance. Throughout this review, we systematically summarized how dysregulated acetylation of histone and non-histone proteins orchestrates multiple hallmarks of glioma biology, including uncontrolled proliferation, diffuse invasion, ferroptosis resistance, glioma stem cell (GSC) maintenance and angiogenesis. Aberrant acetylation patterns, driven by imbalanced activities of lysine acetyltransferases (KATs), histone deacetylases (HDACs), and acetylation readers such as BET family proteins, remodel chromatin architecture, reshape transcriptional networks, and precisely regulate key signaling pathways. Importantly, acetylation acts as a dynamic regulatory interface linking metabolic reprogramming, DNA damage repair and stress adaptation, thereby enabling glioma cells to survive under chemotherapeutic and radiotherapeutic pressure. These findings collectively establish acetylation not merely as a passive epigenetic marker, but as an active driver of glioma aggressiveness and treatment failure.

From a therapeutic perspective, targeting acetylation-related regulatory machinery has shown considerable promise in preclinical and early clinical studies. Inhibitors of KATs (notably p300/CBP), HDACs, and bromodomain-containing proteins effectively suppress glioma growth, impair cancer stemness, and sensitize tumors to temozolomide and radiotherapy in experimental models. Nevertheless, the clinical translation of acetylation-targeted therapies remains challenging. Limited blood–brain barrier (BBB) penetration is a major bottleneck for acetylation-targeted agents in glioma therapy, restricting their translational potential. To address this, several strategies have been developed and validated in preclinical studies. Nanoparticle-based delivery systems (e.g., PLGA nanoparticles, liposomes) protect inhibitors from degradation and enhance BBB crossing *via* transcytosis or receptor-mediated endocytosis. Prodrug approaches modify inhibitor structures to improve lipophilicity and reduce efflux transporter recognition, releasing active drugs in the brain. Rational structural optimization adjusts molecular properties (e.g., lipophilicity, molecular weight) to meet BBB criteria, as seen in CPI-1612 and OTX015. Alternative invasive routes like intrathecal injection and convection-enhanced delivery bypass the BBB to target gliomas directly. These strategies show promise, though further clinical trials are needed to validate their safety and efficacy for translational application. Insufficient target selectivity, off-target toxicities, and intratumoral heterogeneity collectively restrict therapeutic efficacy. Moreover, acetylation-dependent signaling exhibits strong context specificity, cell-type dependence, and extensive crosstalk with other epigenetic and post-translational modifications, including methylation, ubiquitination, phosphorylation, and lactylation. These layers of complexity partially explain why monotherapies targeting single acetylation enzymes often yield modest clinical benefits and highlight the necessity for rational combination strategies.

Looking forward, several directions are critical for advancing acetylation-based glioma therapies. First, the development of subtype-selective and brain-penetrant inhibitors targeting specific KATs, HDAC isoforms, or acetylation readers will be essential to maximize antitumor efficacy while minimizing systemic toxicity. Second, combinatorial treatment strategies integrating acetylation-targeted agents with immunotherapy, targeted therapy, or DNA damage–based modalities warrant intensive investigation to overcome adaptive resistance mechanisms. Third, emerging multi-omics technologies, including acetyl-proteomics, single-cell epigenomics, and spatial transcriptomics, provide unprecedented opportunities to dissect acetylation heterogeneity across glioma subtypes and tumor microenvironments. The identification of robust acetylation-associated biomarkers may enable patient stratification, real-time treatment monitoring, and personalized therapeutic decision-making. Ultimately, a deeper mechanistic understanding of acetylation-centered regulatory networks will facilitate the rational design of precision epigenetic therapies and may open new avenues to improve clinical outcomes for patients with this devastating disease.
